# Case Report: Stable disease achieved in a patient with metastatic castration-resistant prostate cancer following personalized treatment

**DOI:** 10.3389/fonc.2025.1672855

**Published:** 2025-11-26

**Authors:** Fan Zhang, Li Zhang, Dongzi Pang, Shuqing Wei

**Affiliations:** 1 Department of Comprehensive Medicine, Shanxi Province Cancer Hospital/Shanxi Hospital Affiliated to Cancer Hospital, Chinese Academy of Medical Sciences/Cancer Hospital Affiliated to Shanxi Medical University, Taiyuan, China; 2 Department of Urology, Shanxi Province Cancer Hospital/Shanxi Hospital Affiliated to Cancer Hospital, Chinese Academy of Medical Sciences/Cancer Hospital Affiliated to Shanxi Medical University, Taiyuan, China

**Keywords:** castration-resistant prostate cancer, tumor organoids, bicalutamide, personalized management, drug screening

## Abstract

Castration-resistant prostate cancer (CRPC), particularly metastatic castration-resistant prostate cancer (mCRPC), is a leading cause of cancer-related death in men worldwide. Despite great achievements in the treatment of mCRPC, the clinical treatment still faces enormous challenges due to natural or acquired drug resistance. Tumor organoids, an *in-vitro* three-dimensional microstructure, have been demonstrated to predict the response to various therapies to optimize patient outcomes at the individual level. Here, we shared a mCRPC patient who achieved stable disease after treatment with the drugs sensitive in organoid drug screening, despite failure in previous several standard therapies. This typical case highlights that prostate cancer organoids may serve as a potential companion tool to optimize treatment options and improve treatment outcomes, thus realizing the personalized management of mCRPC patients, especially those exhausting the standard therapies.

## 1 Introduction

Castration-resistant prostate cancer (CRPC), particularly metastatic castration-resistant prostate cancer (mCRPC), is one of the most common malignancies and a leading cause of cancer-related death in men worldwide (1). Diverse treatment modalities have been used for mCRPC, mainly including endocrine therapy, chemotherapy, immunotherapy, and bone-targeted therapy. Over the past few years, with the emergence of more novel agents, such as taxanes, poly (ADP-ribose) polymerase (PARP) inhibitors and androgen receptor signaling inhibitors, great achievements have been made in treating mCRPC (2). However, the clinical treatment of mCRPC still faces enormous challenges due to natural or acquired drug resistance.

As *in-vitro* three-dimensional (3D) microstructures from adult or embryonic stem cells with the capability of self-organization and self-renewal, tumor organoids have been confirmed to be a promising model system to enhance translational research and clinical decision-making (3). They can predict the response to various therapies (chemotherapy, radiotherapy, targeted therapy and immunotherapy) to optimize patient outcomes at the individual level (4). Recent studies have demonstrated that prostate cancer organoids can not only evaluate the drug response in clinical trials but also facilitate the study of molecular mechanisms underlying drug resistance and the screening of potential therapies (5, 6). Here, we reported a case of mCRPC who failed in several standard therapies but finally obtained stable disease after treatment with the drugs sensitive in organoid drug screening.

## 2 Case presentation

A 65-year-old man came to our hospital for prostate cancer chemotherapy. He had a previous history of hypertension for 5 years, but no family history of malignancies. In September 2018, the patient developed intermittent dysuria, accompanied by urinary frequency and urgency, which was not paid enough attention. Three months later, he suffered from urinary retention after drinking alcohol, and the symptoms improved following indwelling catheterization.

**Figure 1:**
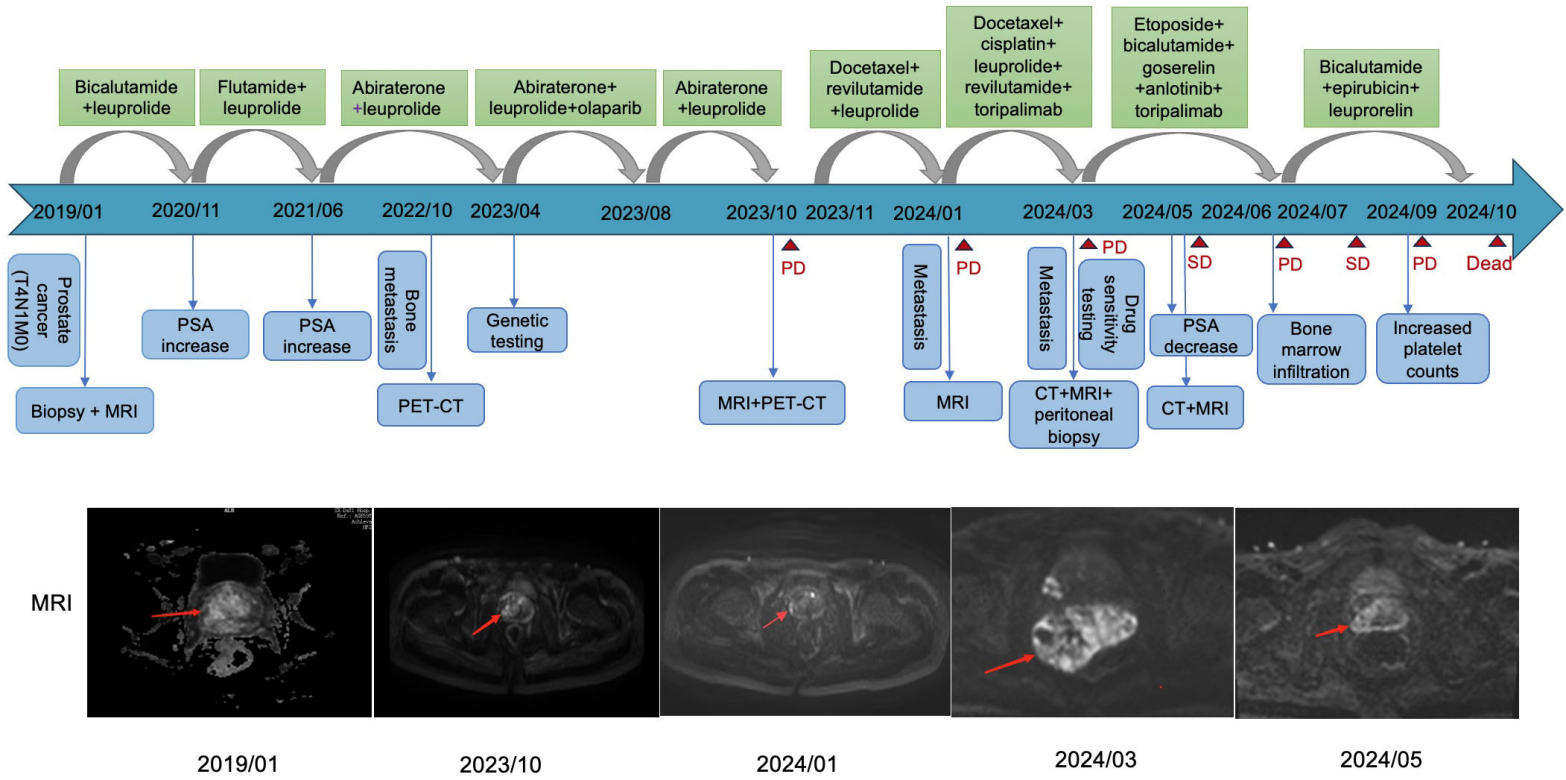
The diagnostic and treatment timeline of the patient.

**Figure 2:**
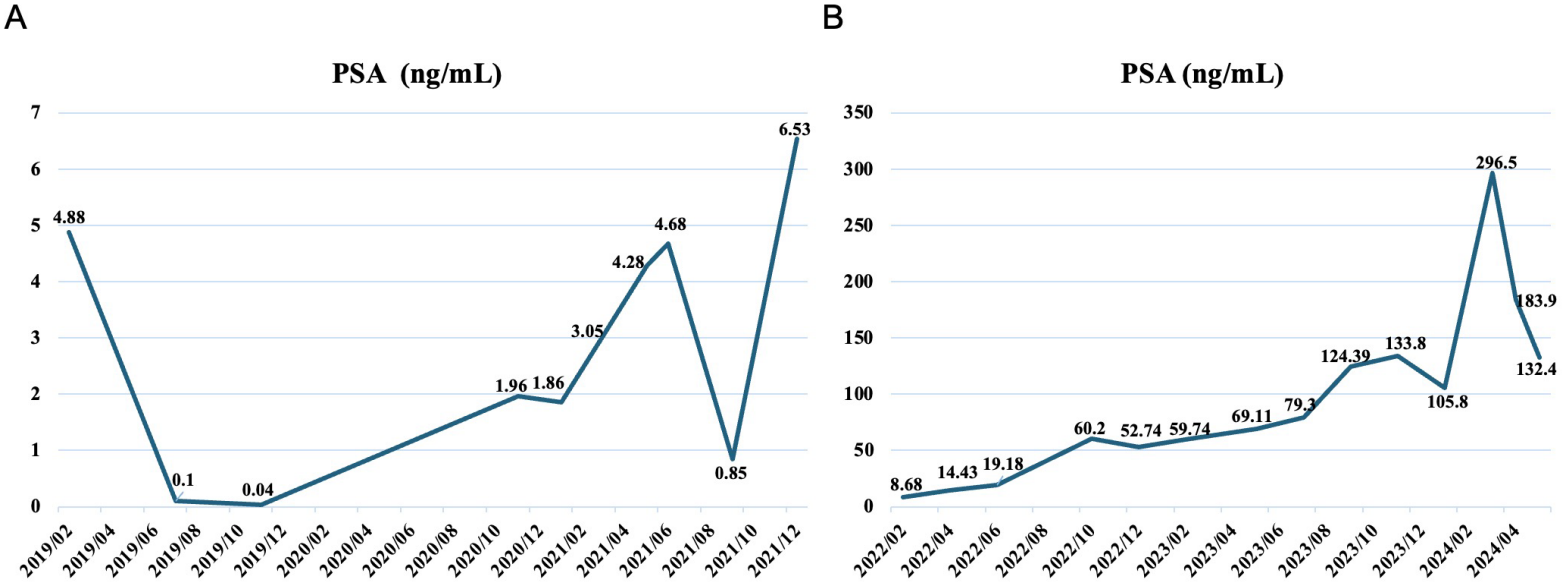
Variations in prostate-specific antigen levels during the treatment from 2019/02 to 2021/12 **(A)** and from 2022/02 to 2024/05 **(B)**.

**Figure 3:**
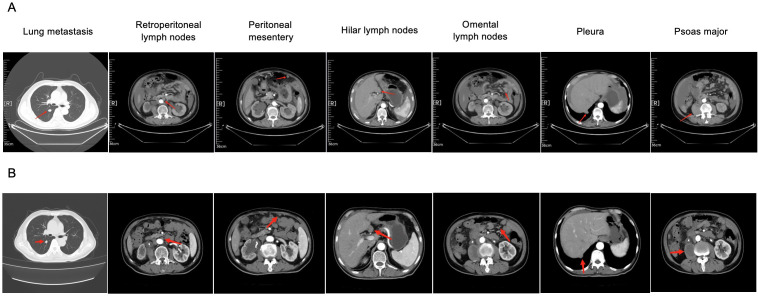
CT scans of the chest and abdomen before **(A)** and after **(B)** the treatment guided by the organoid drug screening. Red arrows head towards to the lesions in different locations.

On January 8, 2019, prostate biopsy showed prostate cancer, Gleason score 4 + 4, in which neural invasion could be observed. Magnetic resonance imaging (MRI) revealed a space-occupying lesion in the prostate that involved seminal vesicle glands and had unclear boundary with the bladder floor, as well as multiple swollen lymph nodes in the pelvic cavity (**Figure 1**). Stage IV prostate cancer (T4N1M0) was considered. Accordingly, bicalutamide plus leuprolide was administrated. In November 2020, the treatment regimen was adjusted to flutamide plus leuprolide due to elevated prostate-specific antigen (PSA) levels from 0.04 to 1.96 ng/mL. In June 2021, the regimen was adjusted again to abiraterone plus leuprolide because of continuously increased PSA levels (**Figure 2A**). In October 2022, positron emission tomography/computed tomography (PET/CT) examination showed multiple bone malignant tumors (MT) and MT lymph nodes in the posterior of peritoneum, by the side of the right iliac vessels and in the intervals between right lateral internal obturator muscles, suggesting bone metastasis. Therefore, bisphosphonates were used for symptomatic treatment. In March 2023, percutaneous drainage was performed due to right hydronephrosis. One month later, somatic mutations in *CHEK2* (c.203_204in sGC and p.Q69fs in nucleotide and amino-acid changes; variant frequency of 0.41%), *TP53* (c.880del and p.E294fs in nucleotide and amino-acid changes; variant frequency of 1.15%), and *KMT2C* (c.2170A>T and p.K724 in nucleotide and amino-acid changes; variant frequency of 0.83%) were identified through genetic testing. *CHEK2* p.Q69fs mutations in homologous recombination repair (HRR) genes suggested that the patient may derive benefits from olaparib, a poly (ADP-ribose) polymerase (PARP) inhibitor. Therefore, olaparib was added for 6 cycles of treatment. On October 26, 2023, PET/CT indicated slightly increased prostate cancer lesions and lymph node metastases than before, suggesting disease progression. Subsequently, the combination of docetaxel, revilutamide and leuprolide was used for 4 cycles. On January 25, 2024, pelvic MRI showed a hyperintense range of the right prostate DWI sequence and some reduced lymph nodes in the right iliac vessels, but newly increased multiple nodules and masses in the left parailiac vessels, peritoneum, and pelvic cavity, thus metastasis was considered. After 2 cycles of treatment with docetaxel, cisplatin, leuprolide, revilutamide and toripalimab, the computerized tomography (CT) scan of the chest and abdomen showed dorsal nodules in the inferior lobe of right lung, bilateral pleural thickening, and peritoneal nodules (**Figure 3A**). Despite no significant changes in prostate cancer lesions, there were enlarged peritoneal and pelvic nodules and masses through the MRI examination on March 24, 2024. Based on the peritoneocentesis, prostate cancer metastasis was further confirmed.

**Figure 4:**
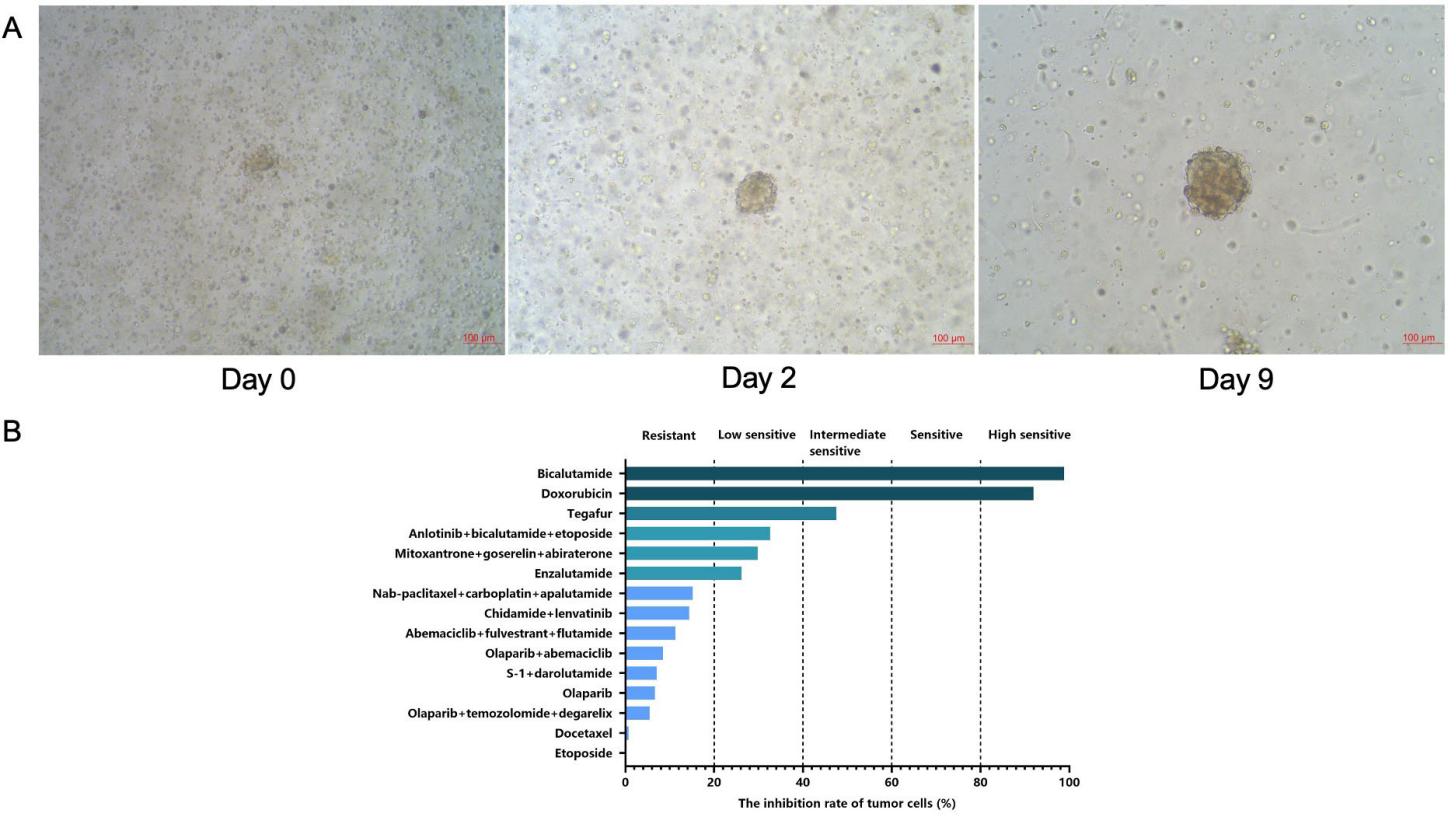
The growth of prostate cancer organoids from malignant ascites under optical microscopes **(A)** and the drug panel for drug sensitivity testing **(B)**.

After the patient provided written consent form, 290 mL of malignant ascites was collected into the heparinized sterile bags by peritoneocentesis and then transferred to the laboratory on ice. Organoid culture and drug sensitivity testing were performed strictly based on the protocols provided by Kingbio Medical Co., Ltd. (Chongqing, China). After centrifugation at 300 g for 5 min at 4°C, the cell pellets were resuspended with Matrigel (354230, Corning), and 40 μL of the suspension was inoculated in 24-well plates at 37°C for 20 min. Meanwhile, 22.5 μL organoid culture medium was added to four unused wells, and 2.5 μL DMSO and 2.5 μL staurosporin solution were added to two unused wells as the negative and positive controls, respectively. Once the droplets were completely solidified, 500 μL of Jiabili^®^ organoid culture medium was added to each well and replaced every three days until the organoids grew like solid spheroids with around 70-μm diameter (**Figure 4A**). Subsequently, drug sensitivity testing was conducted. The established organoids were first digested into single cells using Jiabili^®^ organoid digestive juice, and then Jiabili^®^ tumor tissue basic medium II was utilized for resuspension. The number of organoids were calculated. When Matrigel and Jiabili^®^ organoid medium for prostate cancer were both supplemented, the suspension seeded onto multi-well plates was placed in an incubator at 37°C for 24 hours. Afterwards, the pre-prepared drug solution was added, and the culture for 72 hours was performed. Finally, the number of viable cells in organoids was measured using CellTiter-Glo^®^ Luminescent Cell Viability Assay. There were three replicates for each drug. Totally 15 different drugs were selected, including commonly used chemotherapy drugs and targeted drugs alone or in combination (**Figure 4B**). Notably, the organoids employed in the testing were at passage 1, and the time from sample collection to testing was 20 days. The drug testing concentration depended on the blood drug concentration of each drug, usually ranging from 100 μM to 0.1 nM. Based on the quantification of the inhibitory effects of drugs on tumor organoid growth, a five-classification method was used to evaluate the potential efficacy of the drugs.

During the culture period, the patient was treated with etoposide (100 mg, continuous use of 5 days and then stop use of 16 days), bicalutamide (50 mg, qd), goserelin (3.6 mg, once every 28 days), anlotinib (12 mg, qd, continuous use of 14 days and then stop use of 7 days) and toripalimab (240 mg, once every 3 weeks). Importantly, the patient was found to be highly sensitive to bicalutamide and sensitive to the combination of anlotinib, bicalutamide and etoposide after performing drug sensitivity testing in March 2024 (**Figure 4B**). Considering a trend of PSA reduction from 296.5 to 183.9 ng/mL, this combined regimen continued to be used. Notably, toripalimab was administered empirically, as immune-related biomarkers such as programmed death ligand-1 (PD-L1) expression, tumor mutational burden (TMB), and microsatellite instability (MSI)/mismatch-repair (MMR) status were not available for the patient. On May 17, 2024, grade I neutropenia and hemoglobin adverse events occurred, but the treatment regimen was not adjusted. After 2 cycles of treatment, PSA levels were further reduced to 132.4 ng/mL on May 20, 2024 (**Figure 2B**). Compared with the last examination, MRI showed slightly reductions in prostate cancer lesions and some lymph nodes; CT revealed decreased solid nodules in the dorsal segment of inferior lobe of right lung, improved pleurae, absorbed pleural effusion in the right side, as well as lessened nodes in multiple locations including mediastinum, peritonea and omenta (**Figure 3B**). According to Response Evaluation Criteria in Solid Tumors (RECIST version 1.1), stable disease was assessed. On June 27, 2024, the patient felt weak, thus bone marrow aspiration was performed, indicating bone marrow infiltration. Considering that the patient was highly sensitive to doxorubicin, and epirubicin and doxorubicin were both anthracycline drugs. Compared with doxorubicin, epirubicin showed lower cardiotoxicity and milder myelosuppression. Thus, epirubicin was selected and the treatment regimen was adjusted into bicalutamide, epirubicin plus leuprorelin on July 2, 2024. Unfortunately, the blood platelet counts significantly increased following 2 cycles of treatment. On October 19, 2024, epileptic seizure occurred. Five days later, the patient died due to disease progression.

## 3 Discussion

Prostate cancer, a highly heterogeneous disease whether in molecular or in clinical aspects, may be aggressive due to development of resistance to treatment. It is reported that 10%-20% of prostate cancer patients with metastasis develop CRPC within 5 years, with the median survival of around 14 months after diagnosis of CRPC (7). Although several novel treatment options have been developed for mCRPC, the prognosis is still dismal, and there is also lack of consensus on the timing and sequence of available therapies (8, 9). Recently, tumor organoids, a powerful 3D model system that can faithfully retain histological and molecular features of metastatic tumors including prostate cancer, have been confirmed to be a promising approach for identification of novel treatment strategies and potential biomarkers (5). In this study, we described a mCRPC patient who finally obtained stable disease under the guidance of organoid-based drug screening after failure in several standard therapies, unveiling that prostate cancer organoids could be used to screen the potential drugs and drug combinations for patients exhausting standard therapies to improve the treatment outcome.

In the era of precision oncology, the optimal treatment sequence and combined therapies are designed through predictive molecular signatures. Genomic/transcriptomic signatures that can capture early pathogenic events and CRPC-related gene aberrations have been identified, but progress on the use of these molecular signatures for guiding treatment has been impeded due to lack of accurate preclinical models and appropriate methods to capture tumor heterogeneity at diverse disease states (10). The recently emerging patient-derived tumor organoids can identify the optimal drug combinations for patients eligible for diverse therapies through high-throughput drug screening. In previous studies, prostate cancer organoids have been established for characterizing the subtypes of prostate cancer and screening effective drugs (11, 12). Additionally, mCRPC organoids are also cultured for assessing the efficacy of targeted drugs (13). In our study, the prostate cancer organoids from malignant ascites were established following disease progression. The organoid-based drug sensitivity testing showed highly sensitive to bicalutamide and sensitive to the combination of anlotinib, bicalutamide and etoposide, based on which the combined regimen was used, and the disease was controlled. This finding further supports the potential of patient-derived tumor organoids in guiding the personalized treatment of the individual refractory mCRPC patients.

It has been confirmed that the *CHEK2* gene plays a crucial role in the DNA damage response (DDR) pathway encoding the regulatory kinase CHK2 in the HRR of double-strand breaks (14), and mutations in DDR genes can result in differential responses of prostate cancer to PARP inhibitors (15). In the Expert Consensus on HRR Gene Testing and Variant Interpretation in Prostate Cancer, *CHEK2* has been explicitly classified as one of the HRR genes associated with the treatment efficacy of PARP inhibitors in prostate cancer (16, 17). The PROfound study also indicated that olaparib could significantly improve the clinical outcomes of patients with metastatic CRPC harboring HRR alterations (18). In view of this, olaparib was administrated in our case due to identification of *CHEK2* p.Q69fs mutations, but disease progression still occurred. Notably, HRD or BRCA-like signatures were not assessed. In the subsequent drug sensitivity testing, olaparib was further confirmed unsensitive.

PSA, one of the most extensively used tumor marker, is closely associated with the risk of developing prostate cancer and considered to be the first-line biomarker for the management of prostate cancer (19). In our case, the PSA levels had been decreased significantly from 296.5 to 183.9 ng/mL since the combined regimen with etoposide, bicalutamide, goserelin, anlotinib and toripalimab was used, during which organoid drug sensitivity testing indicated highly sensitive to bicalutamide and sensitive to the combination of etoposide, bicalutamide and anlotinib. Based on this, this combined regimen continued to be used for one more cycle. Continuously decreased PSA levels were observed, and stable disease was assessed based on the MRI and CT examinations. Therefore, for mCRPC patients exhausting standard therapies, patient-derived prostate cancer organoids contributed to identification of effective drugs and drug combinations against tumors through high-throughput screening to improve the treatment outcome. Previous studies also highlighted the ability of tumor organoids to predict clinical responses to therapies, thus determining to tailor therapies timely prior to onset of drug resistance (20). In a recently published expert consensus, organoid-based drug sensitivity testing has been recommended for the different application scenarios of tumors, such as palliative chemotherapy, ineffective first-line treatment, advanced and metastatic cancer, etc., and even can be performed throughout the entirety of cancer treatment (21).

A critical limitation of this study is the failure to detect immune-related biomarkers, including PD-L1, TMB, and MSI/MMR status, which significantly restricts the accurate interpretation of the therapeutic contribution of toripalimab, a PD-1 inhibitor. Although the predictive value of PD-L1 expression in prostate cancer has not yet been fully standardized, it remains an important reference index for evaluating tumor immunogenicity. TMB and MSI/MMR status are directly associated with the ability of tumors to generate neoantigens (22). Lack of such data makes it impossible to identify whether the patient belongs to a potential benefit subgroup. During treatment, the changes in the patient’s condition may be attributed to the pharmacological effects of the PD-1 inhibitor, or may be related to combination therapy, tumor heterogeneity, or individual immune status. Multiple evidence has shown that PD-1 inhibitors have limited efficacy in unselected mCRPC patients (23, 24). Meanwhile, mCRPC generally exhibits a “cold tumor” immune microenvironment, which further reduces the response rate of unselected populations to PD-1 inhibitors. Additionally, there are also several limitations on the clinical application of tumor organoids. First, standardized evaluation criteria and culture protocols are absent. Organoid culture is under the influence of tumor cell activity, cell composition, and tumor heterogeneity. For certain cancer types, such as prostate cancer (25), the low success rate hampers repeatability and reproducibility, consequently affecting high-throughput screening results. Therefore, it should unify the culture conditions and laboratory operations as soon as possible. Second, some *in vivo* components like fibroblasts and immune cells are lacking in tumor organoids, leading to inaccurate recapitulation of the tumor microenvironment. With the development of organoid technology, complicated organoid co-cultures with immune cells, cancer-associated fibroblasts and vasculatures have been developed, and this limitation should be overcome shortly. Additionally, drug concentrations administered to the patients differ from those of acting on tumor cells *in vitro* due to the pharmacokinetics after administration. To reduce *in vitro*-clinical discordances, future organoid models should incorporate pharmacokinetic/pharmacodynamic modeling, such as dynamic drug concentration gradients, and *in vitro* metabolism systems. By addressing these limitations, organoids can better bridge the gap between preclinical research and clinical practice, enabling more reliable personalized treatment decisions.

In conclusion, we described a mCRPC patient who finally obtained stable disease under the guidance of organoid-based drug screening after failure in several standard therapies. This unique case highlights that prostate cancer organoids may serve as a potential companion tool to optimize treatment options and improve treatment outcomes, thus realizing the personalized management of mCRPC patients, especially those exhausting the standard therapies.

## Data Availability

The original contributions presented in the study are included in the article/supplementary material. Further inquiries can be directed to the corresponding authors.
